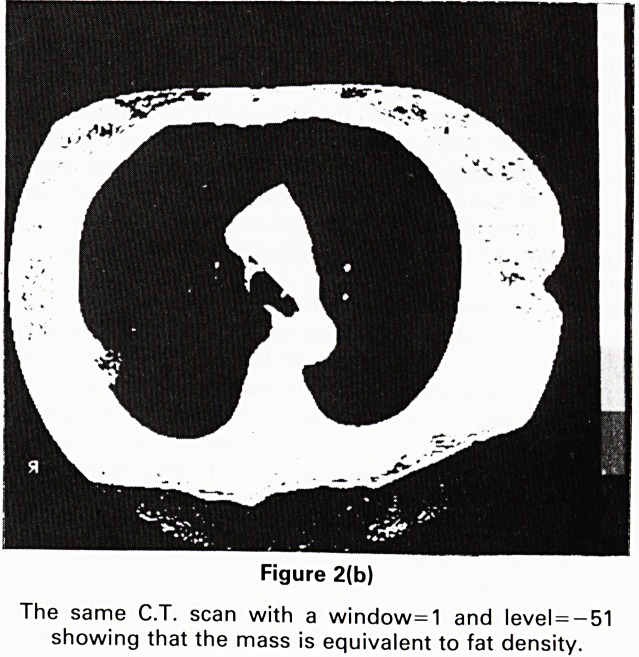# The CT Diagnosis of Pleural Lipoma

**Published:** 1986-04

**Authors:** G. Buirski, P. Goddard

**Affiliations:** Department of Radiodiagnosis, Bristol Royal Infirmary, Bristol; Department of Radiodiagnosis, Bristol Royal Infirmary, Bristol


### Case Report
### The C.T. Diagnosis of Pleural Lipoma

**G. Buirski, M.R.C.P., F.R.C.R.,**
**P. Goddard, M.D., F.R.C.R.,**
Department of Radiodiagnosis, Bristol Royal Infirmary, Bristol

The histological diagnosis of any intra-thoracic mass by non-invasive techniques remains difficult. Those masses lying near the mediastinum may be accessible to bronchoscopy and biopsy whereas peripheral lesions adjacent to the rib cage are more suited to percutaneous needle biopsy.

Computerised Tomography of the thorax provides vital information as to the physical structure and properties of any mass lesion the results of which may enable a histological diagnosis to be made with a reasonable degree of certainty. Its ability to confirm the presence of fatty tissue, however, provides accurate histological proof, thus avoiding the necessity for any further investigations.

We report a case of a peripheral intra-thoracic mass lesion where Computerised Tomography diagnosed the presence of a benign sub-pleural lipoma.

### CASE REPORT

R.H-W. a 71 year old lady was referred to the General Physicians in February, 1984 for control of mild hypertension (170/100). There was no significant past medical history and her clinical examination was normal. A routine chest X-ray (Figure 1) showed a well defined soft tissue opacity adjacent to the right 4th and 5th ribs laterally. A repeat film 3 weeks later was unchanged.

A C.T. scan was performed to exclude the presence of other opacities and to show the primary lesion in more detail. A well defined mass was seen in the posterior part of the right hemithorax, having an attenuation of slightly less than soft tissue.

A similar area of lower attenuation was seen between the lateral aspect of the rib and the serratus anterior (Figure 2). Calculation of the attenuation number confirmed the mass to be of fat density (Figure 3). The diagnosis of a sub-pleural lipoma was made and no further investigations were performed.

**Figure 1**

Chest X-ray showing well defined pleural opacity lying adjacent to the right 5th and 6th ribs laterally.

### DISCUSSION

Intra-thoracic lipomata are rare benign tumours 80% of which arise in the wall of the tracheo-bronchial tree (Fraser and Pare 1976). Other sites include mediastinal sub-pleural and intra-pulmonary and endo-bronchial. The distinction between intra-pleural and intra-pulmonary lesions may only be made by diagnostic

*(Continued on page 43)*

**Figure 2(a)**

C.T. scan showing a mass with an attenuation less than soft tissues extending between the serratus anterior and rib cage laterally and protruding into the thoracic cavity.

**Figure 2(b)**

The same C.T. scan with a window=1 and level=−51 showing that the mass is equivalent to fat density.

### Case Report—The C.T. Diagnosis of Pleural Lipoma *(continued from page 33)*

pneumothorax (Ten Eyck 1960). Sub-pleural lipomata may have a Dumbbell configuration in which part of the tumour protrudes into the thoracic cavity and the remainder is in the intercostal space and beneath the musculature of the chest wall. Confirmation of its extra-thoracic nature is normally made by demonstrating fat within the adjacent soft tissues by plain film tomography. The ability of Computed Tomography to clearly image soft tissues planes and fat makes this modality ideal for the diagnosis of sub-pleural lipomata.

The differential diagnosis of a peripheral intra-thoracic mass lesion seen on a routine chest X-ray is wide. An initial assessment must be made by comparison with previous X-ray films and if the appearances of the lesion are unchanged over a prolonged period of time then this provides important reassurance to both patient and Doctor. If the mass is large enough and accessible, early ultrasound examination may provide useful information. Fluid collections (e.g. empyemas, locculated effusions) are readily diagnosed and may be drained under ultrasound control.

If the lesion is solid, Computed Tomography can assist by showing whether the mass is predominantly extra-thoracic, pleural or intra-pulmonary. Extra-thoracic masses such as the sub-pleural lipoma may now be confidently diagnosed by C.T. Other lesions arising from soft tissue components or the rib cage itself may be clearly identified.

Intra-pleural lesions may be malignant (metastases or mesothelioma) and C.T. may confirm this by demonstrating adjacent soft tissue extension or bone destruction. In many cases it is not, however, possible to distinguish between adenocarcinoma or mesothelioma by C.T. alone (Naidich et al. 1984) C.T. may assist in planning biopsy which can be undertaken by percutaneous needle aspiration, thoracoscopy (endoscopic examination of the pleural space) or by open thoracotomy. A pleural plaque may produce a solitary mass, but the demonstration by C.T. of widespread and often bilateral pleural involvement with or without calcification may indicate a cause such as asbestos exposure (Kreel 1976). Lipomas and fibromas are two uncommon solitary benign pleural tumours, the former of which can be clearly diagnosed by calculation of the attenuation number, but the latter may require biopsy.

### REFERENCES

1. FRASER, R. G. and PARE, J. A. P. (1978) Diagnosis of the diseases of the chest. **II**, p. 995. London, W. B. Saunders Co.
2. KREEL, L. (1976) Computed Tomography in the Evaluation of Pulmonary Asbestosis. *Acta Radiologica Diag.* **17**, p. 405–412.
3. NAIDICH, D. P., ZEHOURIC, E. A. and SIEGELMAN, S. S. (1984) Computed Tomography of the Thorax. p. 259–263. New York, Raven Press.
4. Ten Eyck, E. A. (1960) Subpleural Lipoma. *Radiology* **74**, p. 295–297.

## Figures and Tables

**Figure 1 f1:**
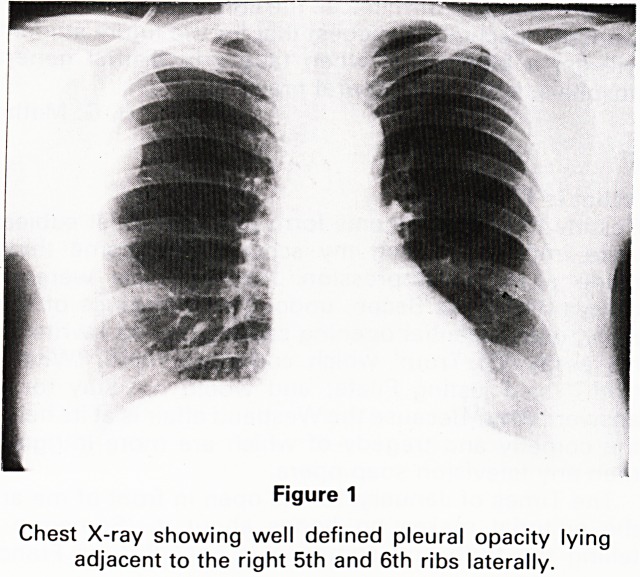


**Figure 2(a) f2:**
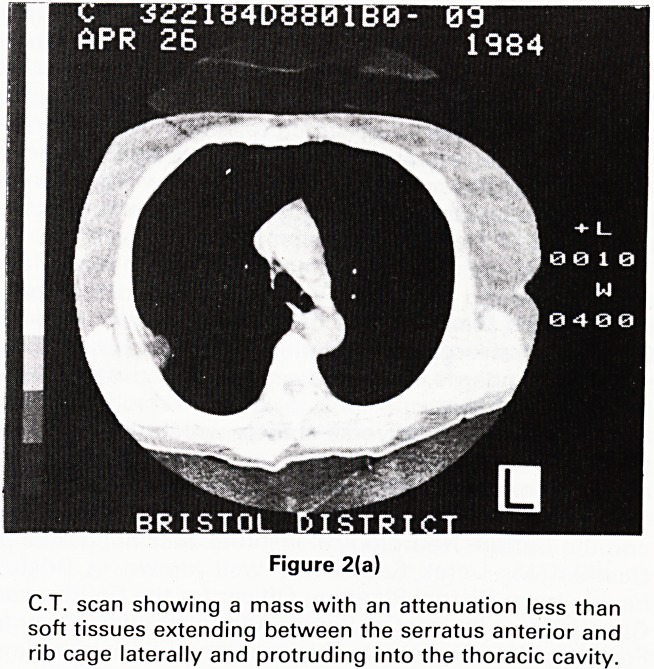


**Figure 2(b) f3:**